# Comprehensive Analysis of Microsatellite-Related Transcriptomic Signature and Identify Its Clinical Value in Colon Cancer

**DOI:** 10.3389/fsurg.2022.871823

**Published:** 2022-03-31

**Authors:** Rui Luo, Yang Li, Zhijie Wu, Yuanxin Zhang, Jian Luo, Keli Yang, Xiusen Qin, Huaiming Wang, Rongkang Huang, Hui Wang, Hongzhi Luo

**Affiliations:** ^1^Department of Colorectal Surgery, The Sixth Affiliated Hospital of Sun Yat-Sen University, Guangzhou, China; ^2^Guangdong Provincial Key Laboratory of Colorectal and Pelvic Floor Diseases, The Sixth Affiliated Hospital of Sun Yat-Sen University, Guangzhou, China; ^3^Department of Tumor Surgery, Zhongshan City People's Hospital, Zhongshan, China

**Keywords:** colon cancer, gene signature, tumor microenvironment, neural network, microsatellite instability

## Abstract

**Background:**

Microsatellite has been proved to be an important prognostic factor and a treatment reference in colon cancer. The transcriptome profile and tumor microenvironment of different microsatellite statuses are different. Metastatic colon cancer patients with microsatellite instability-high (MSI-H) are sensitive to immune checkpoint inhibitors (ICIs), but not fluorouracil. Efforts have been devoted to identify the predictive factors of immunotherapy.

**Methods:**

We analyzed the transcriptome profile of different microsatellite statuses in colon cancer by using single-cell and bulk transcriptome data from publicly available databases. The immune cells in the tumor microenvironment were analyzed by the ESTIMATION algorithm. The microsatellite-related gene signature (MSRS) was constructed by the least absolute shrinkage and selection operator (LASSO) Cox regression based on the differentially expressed genes (DEGs) and its prognostic value and predictive value of response to immunotherapy were assessed. The prognostic value of the MSRS was also validated in another cohort.

**Results:**

The MSI-H cancers cells were clustered differentially in the dimension reduction plot. Most of the immune cells have a higher proportion in the tumor immune microenvironment, except for CD56 bright natural killer cells. A total of 238 DEGs were identified. Based on the 238 DEGs, a neural network was constructed with a Kappa coefficient of 0.706 in the testing cohort. The MSRS is a favorable prognostic factor of overall survival, which was also validated in another cohort (GSE39582). Besides, MSRS is correlated with tumor mutation burden in MSI-H colon cancer. However, the MSRS is a barely satisfactory factor in predicting immunotherapy with the area under the curve (AUC) of 0.624.

**Conclusion:**

We developed the MSRS, which is a robust prognostic factor of overall survival in spite of a barely satisfactory immunotherapy predictor. Further studies may need to improve the predictive ability.

## Introduction

Microsatellite, also known as short tandem repeats (STRs), refers to the DNA motifs, which repeat 50–100 times with 1–6 bases as repetitive units. If mismatch repair system defects, when DNA replicates, one or more bases can be added or deleted from the repetitive units of the substrand, which causes changes in the length of microsatellite sequences (1, 2). Thus, the mutated genes can alter the cell phenotype and may cause disease, even cancer (3). In clinical practice, we use immunohistochemistry to detect mutL homolog 1 (MLH), mutS homolog 2 (MSH2), mutS homolog 6 (MSH6), and postmeiotic segregation increased 2 (PMS2) proteins to reflect the mismatch repair system and if one or more of these proteins cannot be detected or dysfunctioned, it is deficient mismatch repair (dMMR), otherwise proficient mismatch repair (pMMR) (4, 5). Likewise, microsatellite status can be detected by PCR amplification. If two or more of the microsatellite locus (BAT25, BAT26, D2S123, and D5S346) demonstrate microsatellite instability, it would be classified as microsatellite instability-high (MSI-H); if only one locus demonstrates microsatellite instability, it is microsatellite instability-low (MSI-L); otherwise, it is microsatellite stability (MSS) (6–8). MMR is usually equal to MSI-H though sometimes dMMR can be detected, MSI-H cannot be detected in some cases and vice versa (9).

The tumor microenvironment (TME) is an intricate network that is composed of cellular (vascular endothelial cells, fibroblasts, and infiltrating inflammatory cells) and noncellular components (extracellular matrix) and can sustain tumor growth dynamically (10–12). Cancers with microsatellite instability theoretically can produce many neoantigens, which may then be presented to CD8^+^ T cells by histocompatibility complexes (MHCs) molecules (13). So, these cancers may be infiltrated by increased tumor-infiltrative T cells in the TME. However, the cytotoxic T-lymphocyte-associated protein 4 (CTLA-4) and programmed cell death-1 (PD-1) receptors on the surface of the T-cell membrane are hyperactivated, which can help the cancer cells to evade the immune system (14, 15). By blocking these immune checkpoints, the suppressed immune system can be reactivated so that tumor cells can be killed by the immune system and based on this principle, immune checkpoint inhibitors (ICIs) in the recent clinical trials have shown favorable objective response (16, 17) and improved survival outcomes (18) in MSI-H/dMMR metastatic colorectal cancer. Although neoadjuvant/adjuvant ICIs have not been recommended in early-stage colorectal cancer, the use is explored in ongoing clinical trials and case series and showed favorable responses (9, 19). In summary, microsatellite status can serve as an indicator of response to immunotherapy.

Efforts have been devoted to identify more precise biomarkers of response for immunotherapy. Immune checkpoint immunochemistry staining may be the most available method. However, the lack of standardization of these immune checkpoint expressions limits its clinical development (20). Furthermore, neoantigens/mutational burden, immunoscore, and so on have been proposed to be promising prognostic markers (21–24). With the development of high-throughput sequencing, gene expression can be obtained from the tumor samples. Based on this, the gene expression signature is an emerging classification for the response to immunotherapy of colorectal cancer (25–27).

Microsatellite status and gene signature can serve as promising predictors for response to immunotherapy. To the best of our knowledge, no studies have established microsatellite status-related gene signatures to discuss their predictive value for response to immunotherapy in colon cancer. Few studies discussed the prognostic value of microsatellite status-related gene signature in colon cancer, although the previous study has indicated that the microsatellite-related gene signature (MSRS) can predict survival well in gastric cancer (28, 29). Thus, the predictive value of the MSRS for response to immunotherapy is highly anticipated to be established, together with other clinical values (e.g., overall survival, etc.,).

In this study, we analyzed the transcriptional profile and the TME patterns of different microsatellite statuses from publicly available datasets. Then, the MSRS was developed and its prognostic value and predictive biomarker value of response for immunotherapy were evaluated.

## Materials and Methods

### Phenotype and Gene Expression Data

**Figure 1:**
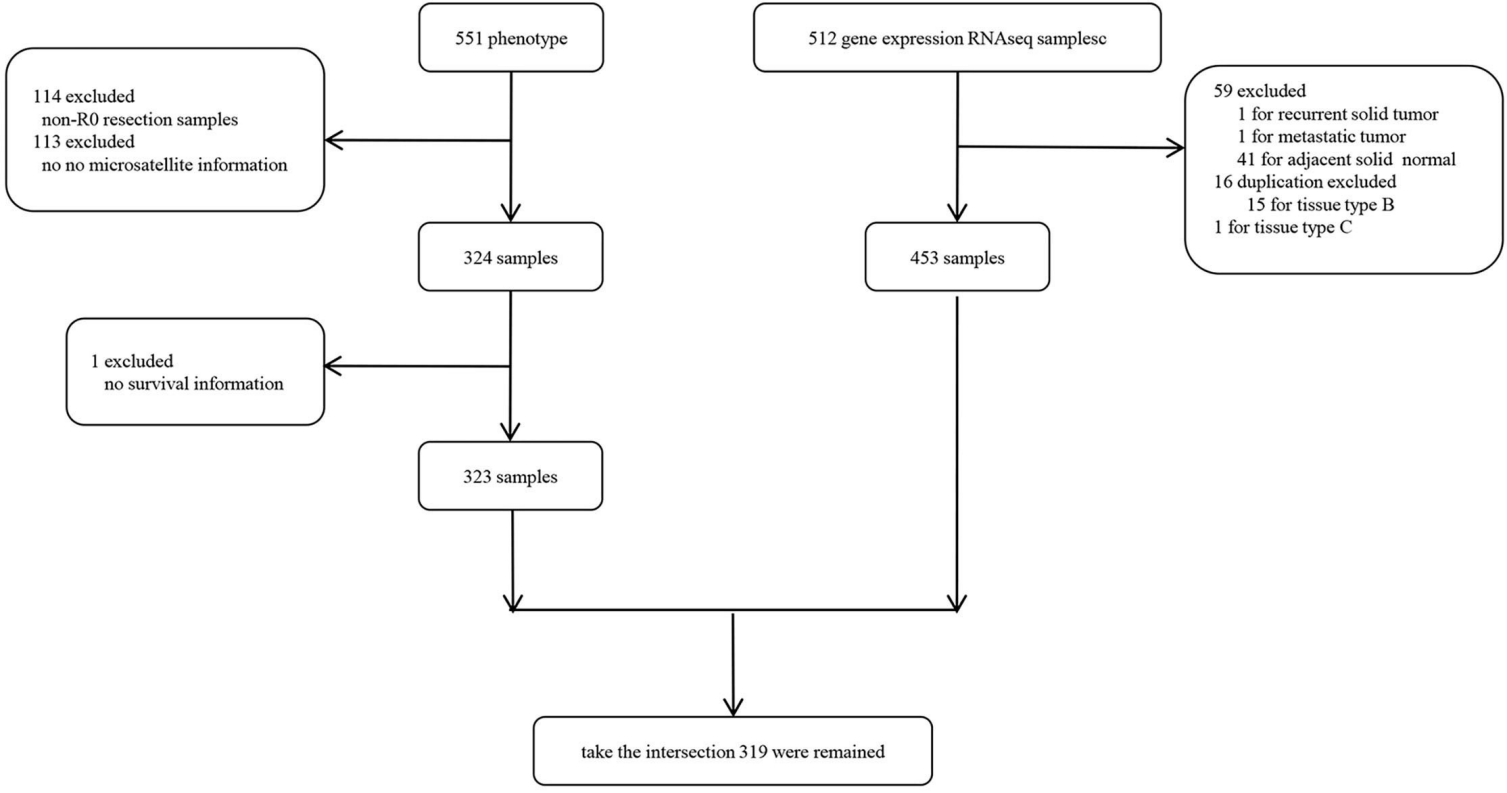
Flowchart of this study.

We obtained the The Cancer Genome Atlas Colon Adenocarcinoma (TCGA-COAD) cohort containing RNA sequencing (RNA-seq) and clinical information from UCSC Xena hub (http://xena.ucsc.edu/public). The gene expression was normalized by fragments per kilobase per million mapped reads (FPKM). Particularly, we only selected the R0 resection samples from the TCGA-COAD to avoid the bias toward survival analysis subsequently and the samples without adequate clinical information were excluded. As shown in Supplementary Figure 1, patients with R0 resection had significantly better survival outcomes even after adjusting for TNM staging. The sample selection flowchart is shown in Figure 1 and a total of 318 samples were finally included in this study.

### Single-Cell RNA-seq and RNA-seq Analysis

The single-cell RNA-seq data and bulk RNA-seq data were used to illustrate the heterogeneity between MSI-H and MSI-L/MSS colon cancer. The single-cell RNA-seq transcriptome metadata of GSE146771 were obtained from the Gene Expression Omnibus (https://www.ncbi.nlm.nih.gov/gds/), which were visualized by the two-dimensional t-distributed stochastic neighbor embedding (t-SNE) method. The “map” package (30) in R was used to dimension reduction and visualize the transcriptome profile by two-dimensional uniform manifold approximation and projection (UMAP) plot for the RNA-seq data included in this study from the TCGA-COAD cohort.

### Immunity Quantification

We used the Estimation of STromal and Immune cells in MAlignant Tumors using Expression data (ESTIMATE) algorithm by “estimate” package to infer the fraction of stromal and immune cells of each sample (31). Besides, we performed single sample gene set enrichment analysis (ssGSEA) to evaluate the infiltrative immune cell enrichment score by the “GSVA” package. The hallmark gene set was retrieved from a published article (32).

### Differentially Expressed Genes (DEGs)

The DEGs were identified by the “limma” package after “voom” normalization (33). Based on the criteria of |logFC| >2 and an adjusted *P*-value < 0.05, the *P*-value was adjusted by false discovery rate (FDR).

### Establishment and Validation of the MSRS

The least absolute shrinkage and selection operator (LASSO) Cox regression, which is popular in dimension reduction for high-dimensional data, was utilized to build the MSRS by using the DEGs. The LASSO Cox regression adds a penalty to the coefficients to shrink the regression coefficients to zero. We determined the penalty parameter λ by 10-fold cross-validation. This was processed by the “glmnet” package. The $\text{MSRS}={\text{Σ}}_{1}^{\text{n}}(Expr_{i}\times Coef_{i}),$, where the Expr_i_ represents the gene expression value and the Coef_i_ represents the coefficient corresponding to the gene which is determined when the partial likelihood deviance is the least in the LASSO Cox regression. The discrimination power of the MSRS was evaluated by the time-dependent receiver operating characteristic curve together with age, gender, and Tumor, node and metastasis (TNM) staging, which was processed by the “timeROC” package. To determine the impact of the MSRS on overall survival, the Kaplan–Meier (KM) survival analysis was used. In addition, the effect of the MSRS on overall survival was evaluated by the multivariate Cox regression analysis to adjust for other clinical covariates. The KM survival analysis and the multivariate Cox regression analysis were performed by the “survival” and “survminer” package. In addition, the prognostic value of the MSRS was validated in the external GSE39582 cohort.

### Tumor Mutation Burden Acquisition

We obtained the gene mutation information of the TCGA-COAD from the “TCGAmutations” package (34). To calculate the tumor mutation burden, we assume that the whole exome size is 38 Mb as indicated by a study of Chalmers et al. (35).

### Response to Immunotherapy and Cisplatin

The tumor immune dysfunction and exclusion (TIDE) algorithm was utilized to evaluate the response to immunotherapy (http://tide.dfci.harvard.edu). This algorithm can predict immunotherapy response by pretreatment transcriptomic profiles. We set 1.0 as the cutoff of responder and nonresponder arbitrarily. The “pRRophetic” package was used to predict the response to cisplatin by gene expression matrix (36). We set 4.3 as the cutoff of responder and nonresponder.

## Results

### Single-Cell RNA-seq and RNA-seq Analyses Demonstrate the Heterogeneity Between MSI-H and MSI-L/MSS Colon Cancer

**Figure 2:**
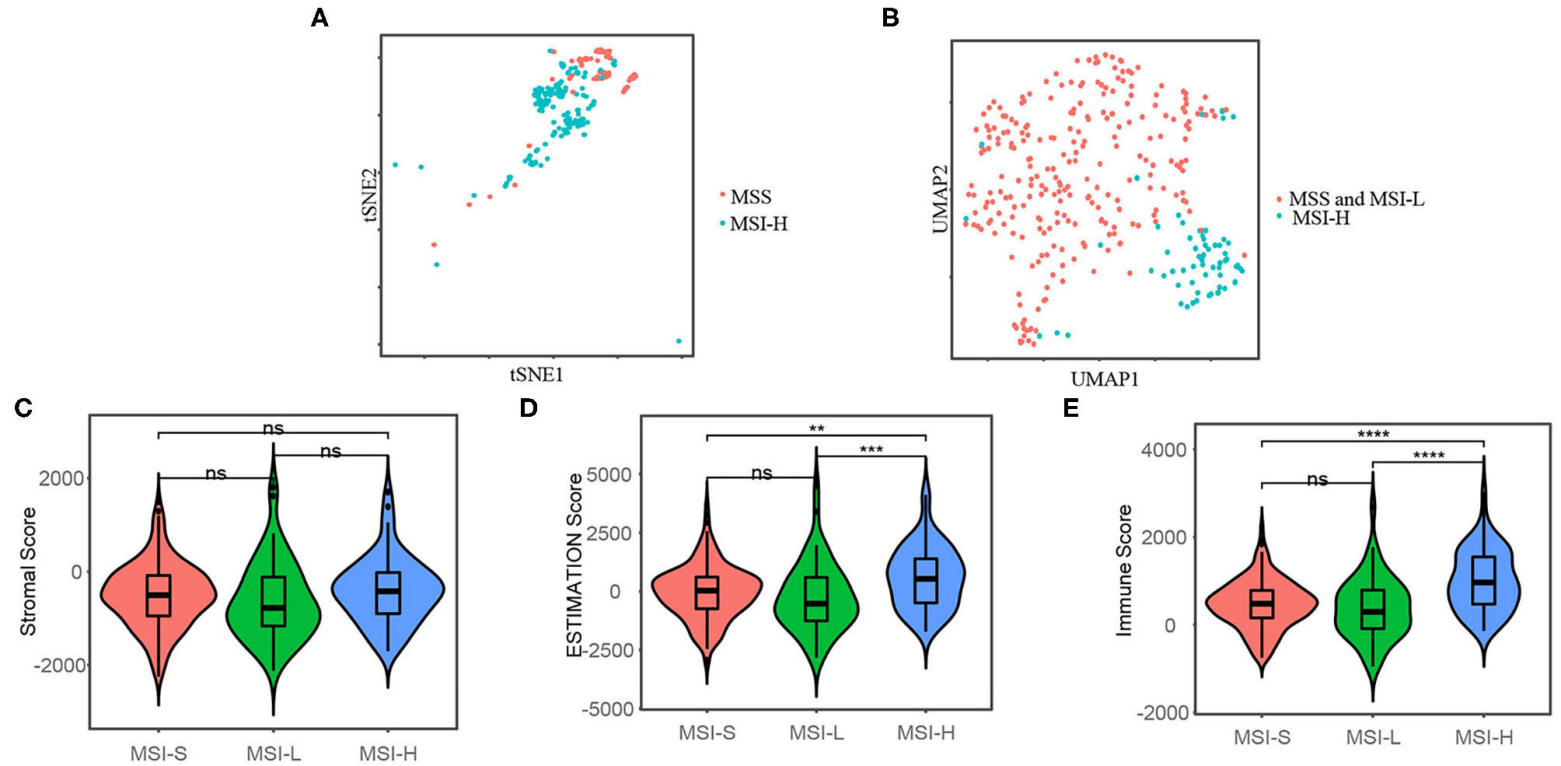
t-distributed stochastic neighbor embedding (t-SNE) plot **(A)** shows that the microsatellite instability-high (MSI-H) and microsatellite stability (MSS) colon cancer cells are clustered differentially by single-cell RNA sequencing (RNA-seq) data from GSE146771 and uniform manifold approximation and projection (UMAP) plot **(B)** shows that the MSI-H and MSS/microsatellite instability-low (MSI-L) colon cancer samples are clustered differentially included in this study from the TCGA-COAD cohort. Stromal score **(C)**, the Estimation of STromal and Immune cells in MAlignant Tumors using Expression data (ESTIMATE) score **(D)**, and immune cell score **(E)** of the different microsatellite status, which represent the stromal cell fraction, tumor purity, and immune cell fraction of tumor samples, respectively. The Mann–Whitney *U-test* was applied for difference comparison between the two groups. The Mann–Whitney *U-test*; ns: *p* > 0.05; *P < 0.05; **P < 0.01; ***P < 0.001; ****P < 0.0001.

To analyze the heterogeneity between MSI-H and MSS/MSI-L colon cancers at the single-cell level, we obtained the single-cell RNA-seq data (GSE146771) from the Gene Expression Omnibus (https://www.ncbi.nlm.nih.gov/gds/). Then, we visualize the transcriptome profile by t-SNE plot, which is commonly used in dimension reduction and visualization for single-cell transcriptome data (37, 38). The microsatellite status information of the single-cell transcriptome data was obtained from the published elsewhere (39). As shown in Figure 2A, the MSI-H and MSS colon cancer cell were clustered differentially. Additionally, we then cluster the samples included in this study from the TCGA-COAD cohort by two-dimensional UMAP, as shown in Figure 2B, which indicated that the MSI-H and MSS/MSI-L samples were clustered differentially.

### Microsatellite Status Shows Different Tumor Immune Microenvironment

**Figure 3:**
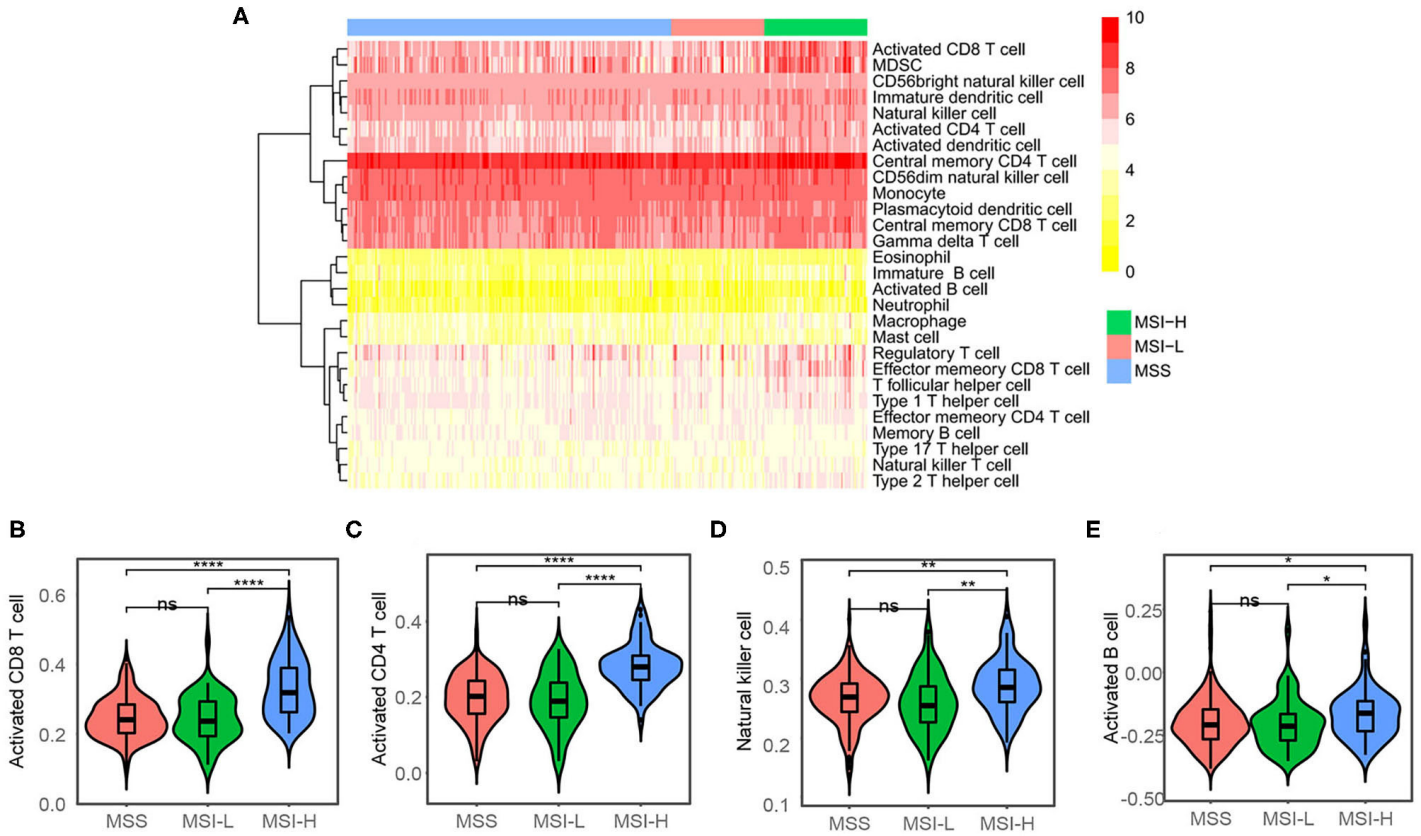
Single sample gene set enrichment analysis (ssGSEA) analysis. **(A)** Heatmap showing the proportions of 28 types of immune cell. The proportions have been normalized to 0–10 across the 318 samples. **(B)** Activated CD8^+^, **(C)** activated CD4^+^, **(D)** natural killer cell, and **(E)** activated B cell were evaluated in the MSI-H samples compared with the MSS/MSI-L samples. ns: *P* > 0.05; *P < 0.05; **P < 0.01; ***P < 0.001; ****P < 0.0001.

We used the ESTIMATE algorithm to infer the fraction of stromal and immune cells to further investigate the immune microenvironment difference. As shown in Figures 2C,D, the MSI-H samples have a larger proportion of immune cells compared with the MSS/MSI-L samples, but not stromal cells. To further explore the populations of 28 types of the immune cell for each sample, we performed ssGSEA as shown in Figure 3A. We found that the MSI-H samples have an evaluated level of activated CD8^+^, activated CD4^+^, natural killer (NK) cell, and activated B cell (Figures 3B,E) and most of the immune cells, except for CD56 bright NK cell (Supplementary Figure 2).

### Construction of a Neural Network to Predict Microsatellite Statuses

**Figure 4:**
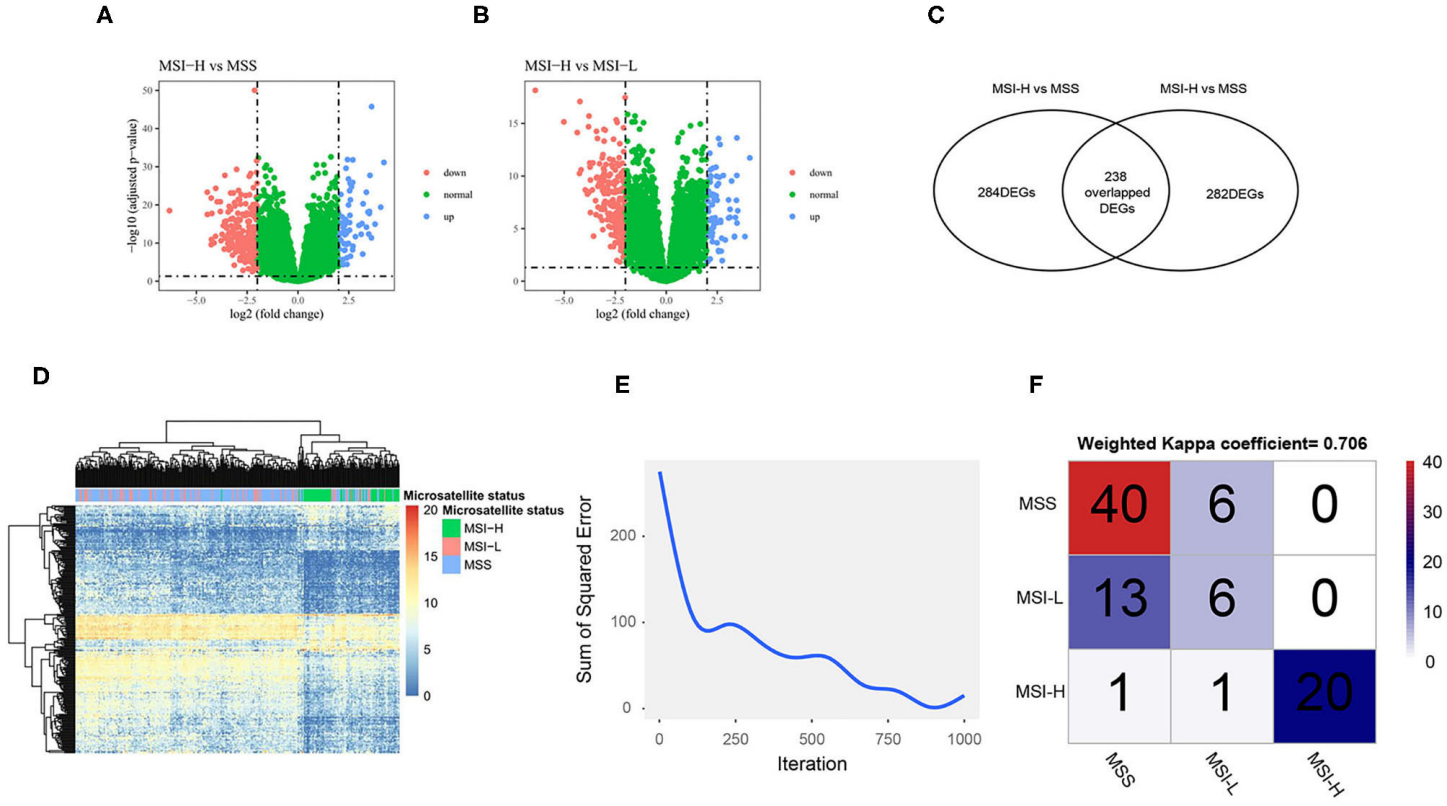
The differentially expressed genes (DEGs) analysis and neural network construction. Volcano plot shows the DEGs of MSI-H vs. MSS **(A)** and MSI-H vs. MSI-L **(B)**; Venn plot shows that 284 DEGs were found between MSI-H and MSS and 282 DEGs were found between MSI-H and MSI-L **(C)**. A total of 238 overlapped DEGs were selected as the final DEGs; heatmap of the DEGs **(D)** shows that the MSI-H samples were clustered differently from the MSS and MSI-L samples. **(F)** The sum of the squared error decreases as the iteration arises and the confusion matrix **(F)** shows the performance of the neural network in the test set.

We performed the DEGs analysis after filtering low expression genes (Supplementary Figure 3) between MSI-H and MSS/MSI-L samples as shown in the volcano plot (Figures 4A,B). The Venn diagram (Figure 4C) shows that with the criteria of |logFC| > 2 and an FDR < 0.05, 284 and 282 DEGs were identified between the MSI-H and MSS/MSI-L samples, respectively. The heatmap of the DEGs (Figure 4D) showed that the MSI-H sample was clustered differently from the MSI-L/MSS samples, which indicates the different expression profiles of the DEGs among different microsatellite statuses. To further explore the association between the microsatellite-related DEGs and microsatellite status, we constructed a neural network with 3 hidden layers of 100, 50, and 25 neurons, respectively. 0.7 of the sample was randomly split into the training set to construct the neural network and the remaining samples were used to test the model. As shown in Figure 4E, as the iteration arises, the sum of the squared error decreases, which indicates that the predictive performance of the neural network model arises as the iteration arises. When tested in the test set, the model showed substantial performance with a Kappa coefficient of 0.706 (Figure 4F).

### Microsatellite-Related Genes Risk Has a Favorable Prognostic Value on Overall Survival

**Figure 5:**
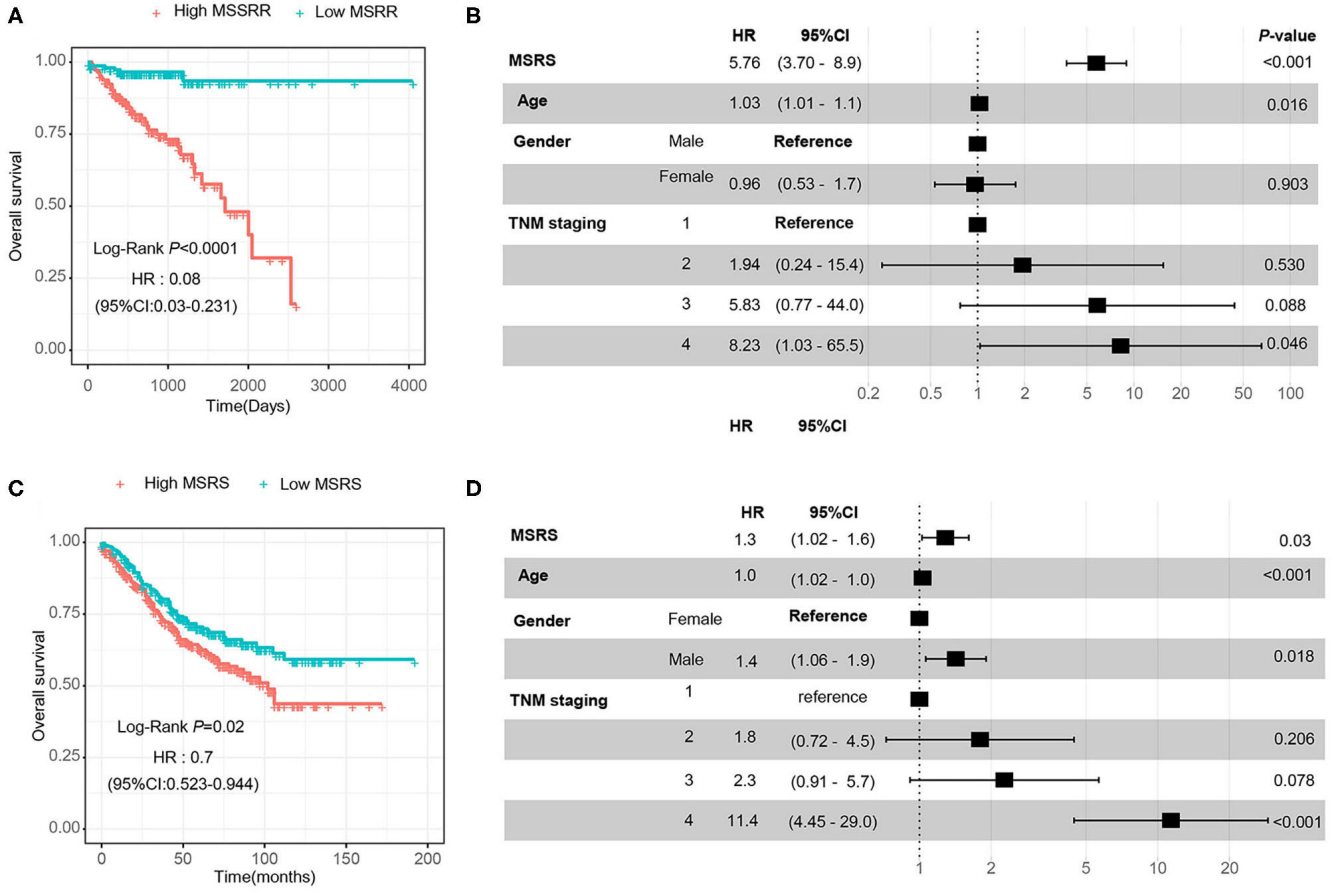
Construction of the microsatellite-related gene signature (MSRS) and its discrimination ability. We selected the penalty parameter when the partial likelihood deviance is the least by 10-fold cross-validation **(A)** and 24 nonzero coefficient genes were remained **(B)**. The waterfall plot of the MSRS **(C)** ranking from smallest to largest. The time-dependent receiver operating characteristic (ROC) curves **(D)** indicate that the MSRS has favorable discrimination power even over TNM staging.

To construct a microsatellite-related genes risk and explore its prognostic value, the LASSO Cox regression was used. We selected the optimal penalty parameter (0.02365558) by 10-fold cross-validation when the partial likelihood deviance is the least (Figure 5A). Then, 24 genes with nonzero coefficients were remained (Figure 5B) and were selected to construct the MSRS (Figure 5C). The coefficients and corresponding genes can be found in Supplementary Table 1. The MSRS showed favorable discrimination on overall survival with the time-dependent area under the curves (AUCs) over 0.8 most of the time (Figure 5D). Besides, compared with other clinicopathological factors, such as age, gender, and even TNM staging, the MSRS showed superior discrimination ability.

**Figure 6:**
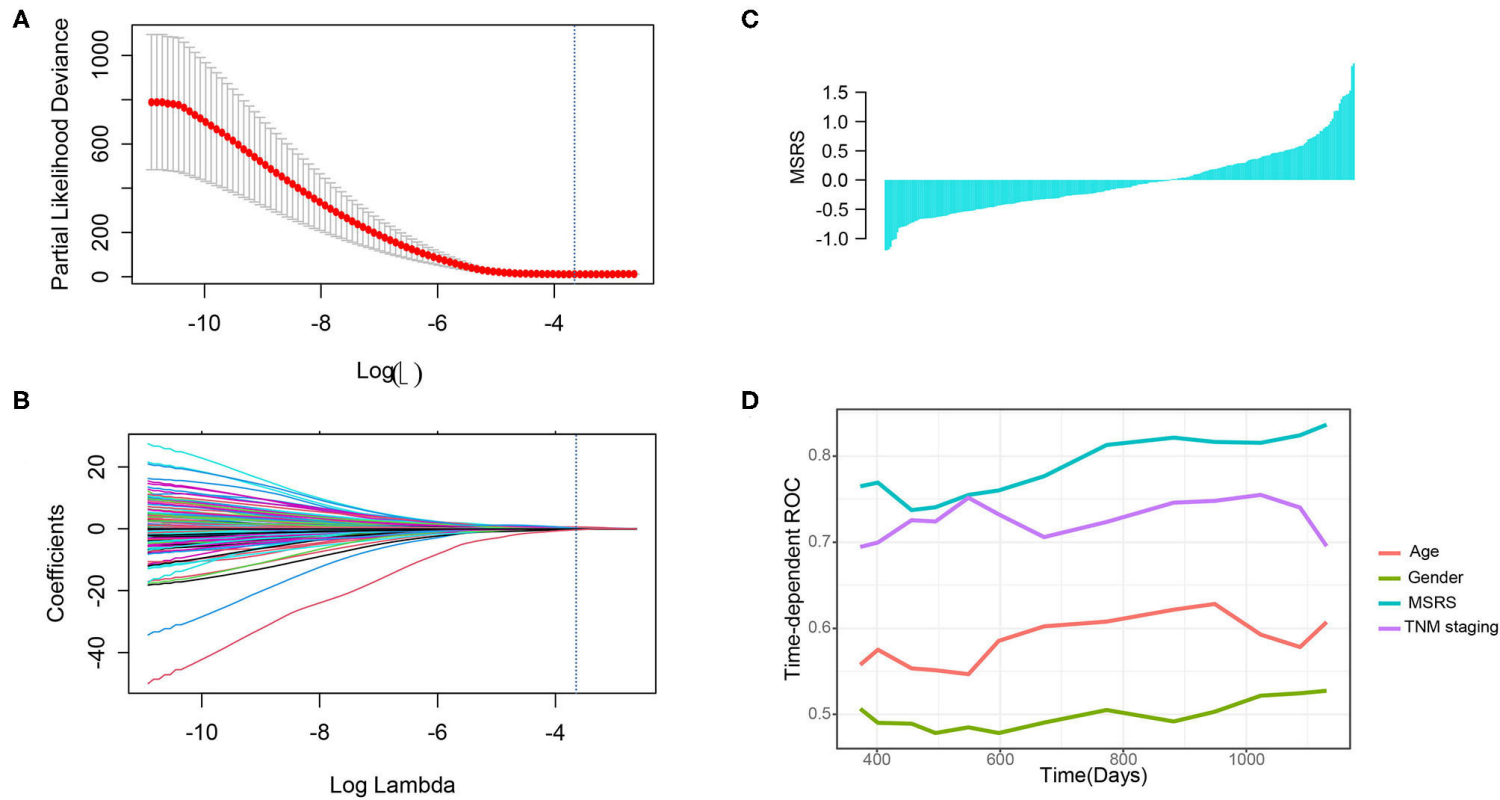
The MSRS has favorable predictive value and validation in the GSE39582 cohort. The Kaplan–Meier (KM) curves **(A)** indicate that the higher MSRS has poor overall survival and it is indicated in the forest plot **(B)**. We validated the prognostic value in the external cohort (GSE39582) and the KM curves **(C)** and the forest plot **(D)** showed similar results.

Then, we divided the MSRS into the high MSRS and the low MSRS via the median MSRS. As shown in Figure 6A, low MSRS had significantly less risk of overall survival and even after adjusting for age, gender, and TNM staging, the MSRS also showed an independent prognostic factor of overall survival (Figure 6B). In the validation cohort (GSE39582), the high MSRS was also a poor prognostic factor and was an independent prognostic factor of overall survival (Figures 6C,D).

### Correlation Between the MSRS and Tumor Mutation Burden per Microsatellite Status

**Figure 7:**
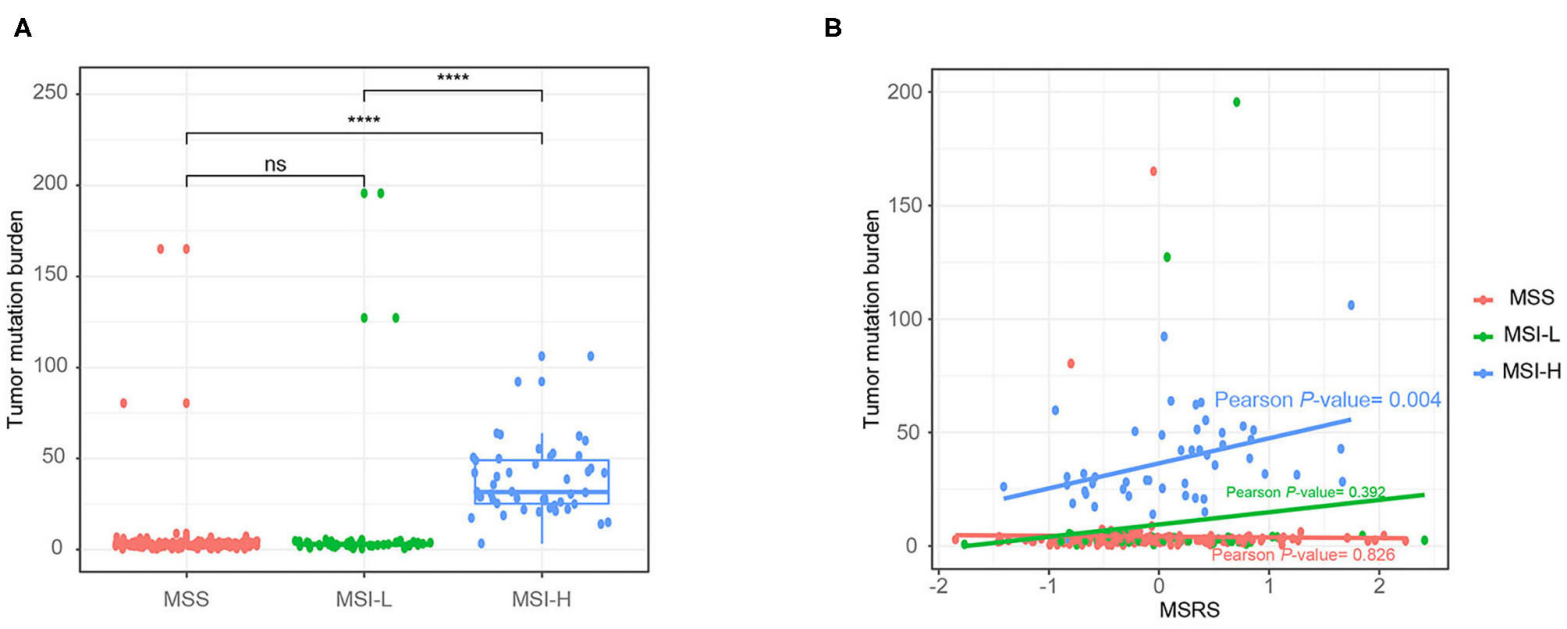
Boxplot showing number of gene mutations against microsatellite status **(A)** and the association between number of gene mutations and the MSRS Mann–Whitney *U-*test **(B)**; ns: *p* > 0.05; *P < 0.05; **P < 0.01; ***P < 0.001; ****P < 0.0001.

In addition, we analyzed correlations between the MSRS and the total number of gene mutations. As shown in Figure 7A, the MSI-H cancers had a significantly higher number of gene mutations than MSS and MSI-L cancers. Besides, in MSI-H cancers, the MSRS was positively correlated with gene mutations with a statistically significant level (Figure 7B, Pearson's *P*-value = 0.004).

### Associations Between the MSRS and Response to Immunotherapy and Cisplatin

**Figure 8:**
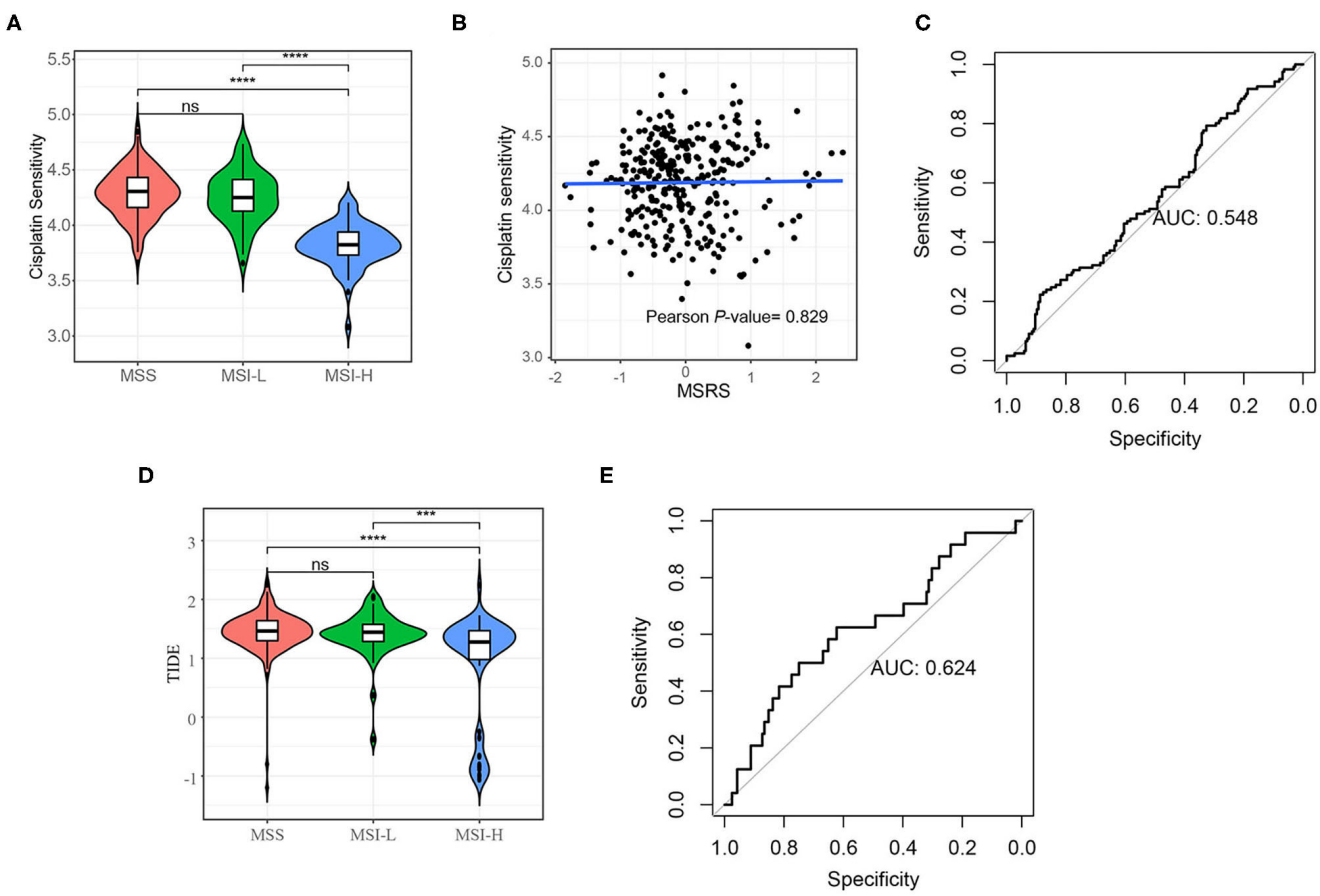
Cisplatin sensitivity and immunotherapy sensitivity analysis. **(A)** Violin plot shows that the MSI-H colon cancers are less sensitivity to cisplatin **(A)** and more sensitivity to immunotherapy **(D)**; **(B)** Scatter plots and the fitting curve show the correlation between cisplatin and the MSRS; **(C)** the ROC curve shows the discrimination ability of the MSRS on cisplatin response **(C)** and immunotherapy response **(E)**. The Mann–Whitney *U-test*; ns: *p* > 0.05; *P < 0.05; **P < 0.01; ***P < 0.001; ****P < 0.0001. AUC: area under the curve.

The predictive value of the MSRS for cisplatin was also analyzed. As shown in Figure 8A, the MSI-H cancers were less sensitive to cisplatin. Unexpectedly, the MSRS was not correlated with cisplatin sensitivity (Pearson's *P*-value = 0.829). The MSRS was not a fairly good marker for cisplatin sensitivity (Figure 8C, AUC = 0.548). Not surprisingly, the MSI-H cancers were more sensitive to immunotherapy (The bigger TIDE is, the more sensitivity is), as depicted in Figure 8D. The predictive value of the MSRS for immunotherapy was barely satisfactory with the AUC of 0.624 (Figure 8E).

## Discussion

In this study, we found that the MSRS could serve as a favorable prognostic factor for overall survival, but a barely satisfactory factor of response to immunotherapy of colon cancer. By merging the DEGs identified from different microsatellite statuses, a total of 238 DEGs had remained, which could well predict the microsatellite status through our neural network. Then, the MSRS was established by the LASSO Cox regression. The MSRS was a well prognostic factor with the favorable time-dependent AUC, which is even better than TNM staging system. Similarly, a previous study showed that the MSRS was also a well prognostic factor with the higher AUCs compared with TNM staging system (40). In this study, patients with the high MSRS remarkably had poor overall survival than with the low MSRS in the TCGA-COAD cohort, even after adjusting for other clinicopathological factors. Besides, this conclusion was also validated in another cohort (GSE146771), which further illustrated the prognostic value of the MSRS. Except for the prognostic value of overall survival, the MSRS is also significantly correlated with tumor mutation burden. Previous studies had demonstrated that tumor mutation burden is a positive prognostic factor for metastatic colorectal cancer patients receiving first-line chemotherapy plus bevacizumab or cetuximab (41) and is a biomarker for the response for ICIs in metastatic colorectal cancer (41). As for early-stage colorectal cancer, tumor mutation burden can also predict the survival prognosis of colorectal cancer treated by curative surgery followed by adjuvant fluoropyrimidine and oxaliplatin chemotherapy (42). These results suggested that the MSRS, which is representative of microsatellite status, is capable of improving prognostic value compared to traditional clinicopathological factors and is associated with other clinical risk factors in colon cancer. However, the MSRS had limited value in predicting response to immunotherapy with the AUC of 0.624 after setting 1.0 as the cutoff TIDE score to divide into responder and nonresponder. Currently, there is no consensus on setting the optimal cutoff value of the TIDE for responder and nonresponder. Further studies are needed to improve the predictive value of the MSRS, which may be realized in other ways such as random forest model and machine learning model. In addition, the TIDE algorithm may not work on colorectal cancer, as indicated, which may confuse the explanation. Not surprisingly, MSI-H colon cancer was less sensitive to cisplatin. Microsatellite status had been a prognostic factor and an indication for chemotherapy in stage II colon cancer and chemotherapy may impair the survival of MSI-H/dMMR cancers (43, 44). The mechanism is still not clear with one assumption of the side effects of chemotherapy that may impair the patient without potentially killing cancer cells. However, the MSRS seemed to not correlate with cisplatin sensitivity and the predictive value of cisplatin response was poor with the AUC of 0.548. Likewise, there is no consensus on which is the optimal cutoff value for cisplatin responder and nonresponder and we set 4.3 as the cutoff value arbitrarily. Any conclusions should be drawn cautiously from this result.

Different transcriptome profiles of different microsatellite statuses were revealed by dimension reduction plots of single-cell RNA-seq and RNA-seq data. This result indicated that MSI-H colon cancers have different gene expression patterns. Not just focusing on tumor cells, the TME also plays an important role in tumor growth, invasion, metastasis, etc., (45). Besides, the response to immunotherapy relies on the dynamic interactions between the tumor cells and the TME (46). The lactate, glucose, and cancer-associated fibroblasts can inhibit T-cell function through certain pathways and/or chemokines secretion, which results in immune tolerance (47). ICIs target the key inhibitory receptor at the core of the dynamic interaction network. In this study, we estimated the immune cells via the ESTIMATION algorithm by RNA-seq data. The immune and stromal scores represent the stromal and immune cell proportions. These results revealed that MSI-H cancers have a higher proportion of immune cells. Specifically, activated CD8^+^, CD4^+^ T cell, NK cell, etc., were observed more in the TME of MSI-H cancers, except for CD56 bright NK cell, which produces great levels of cytokine (48). We believe that with the advances of sequencing method such as spatial transcriptome, understanding the tumor immune microenvironment and immunotherapy response of different microsatellite statuses in colon cancer may be facilitated.

This study also has some limitations. First, the MSRS was established on RNA-seq data, which cannot be routinely used in the clinic. But, the signature based on immunochemistry staining may be developed. Besides, the gene expression was detected by different platforms, which need standardization and the tumor immune microenvironment was estimated *in-silico*, which may generate knowledge gap. To solve this issue, single-cell RNA-seq may be an alternative. As for the clinical information, many survival data in the TCGA-COAD cohort were censored, which may impair the survival curve.

In conclusion, we analyzed the transcriptome profiles and tumor cell infiltration of different microsatellite statuses and constructed the MSRS, which is a favorable survival prognostic factor and has underlying prognostic value in predicting immunotherapy. These findings helped us to better understand the molecular natures of different microsatellite statuses and may provide practical guidance of immunotherapy for patients with colon cancer.

## Data Availability Statement

Publicly available datasets were analyzed in this study. This data can be found here: http://xena.ucsc.edu/public.

## Author Contributions

RL, HW, RH, and HL conceived this study. RL and YL retrieved the data, did the bioinformatics analysis, and drafted this manuscript. KY, XQ, HW, RH, and HL revised this manuscript. All the authors discussed this manuscript and approved the final version of this manuscript.

## Funding

This study was supported by the Sun Yat-Sen University Clinical Research 5010 Program (Grant Numbers 2017008 and 2019021).

## Conflict of Interest

The authors declare that the research was conducted in the absence of any commercial or financial relationships that could be construed as a potential conflict of interest.

## Publisher's Note

All claims expressed in this article are solely those of the authors and do not necessarily represent those of their affiliated organizations, or those of the publisher, the editors and the reviewers. Any product that may be evaluated in this article, or claim that may be made by its manufacturer, is not guaranteed or endorsed by the publisher.
