# Supplementary material for: Comprehensive Analysis of Microsatellite-Related Transcriptomic Signature and Identify Its Clinical Value in Colon Cancer
Source: Front Surg. 2022 Mar 31;9:871823. doi: 10.3389/fsurg.2022.871823 (PMC9008782; doi:10.3389/fsurg.2022.871823)
Supplement: Supplementary file 1 [file Data_Sheet_1.pdf]

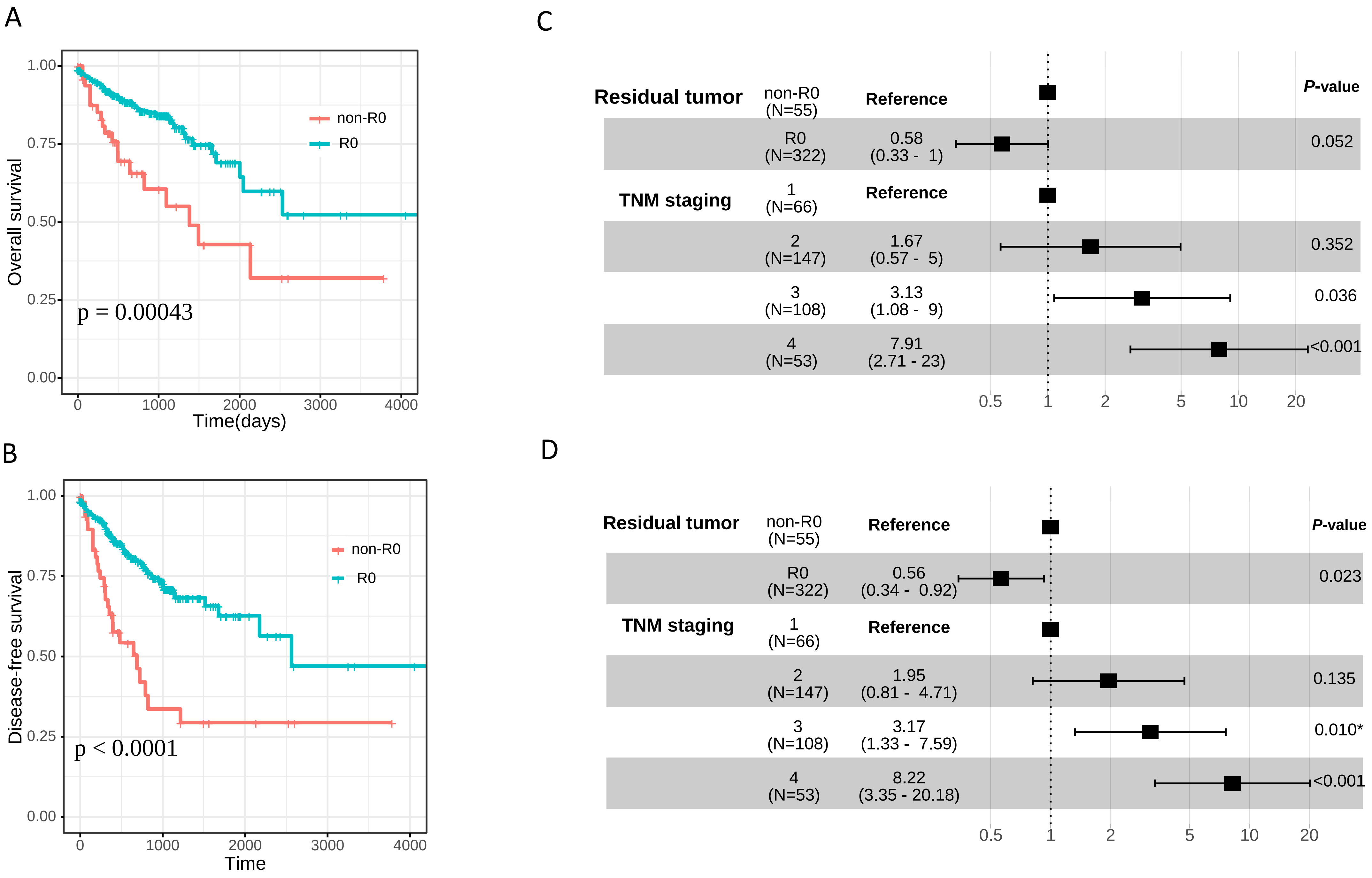

Figure S1. Kaplan-Meier curves show R0 resection samples have a favorable overall survival (A) and disease-free survival (B) and showes less hazard rate compared with non-R0 resection samples even after adjusting for TNM staging in the forestplot(C, D). Although it is not statistically significantly in the overall survival analysis(C)

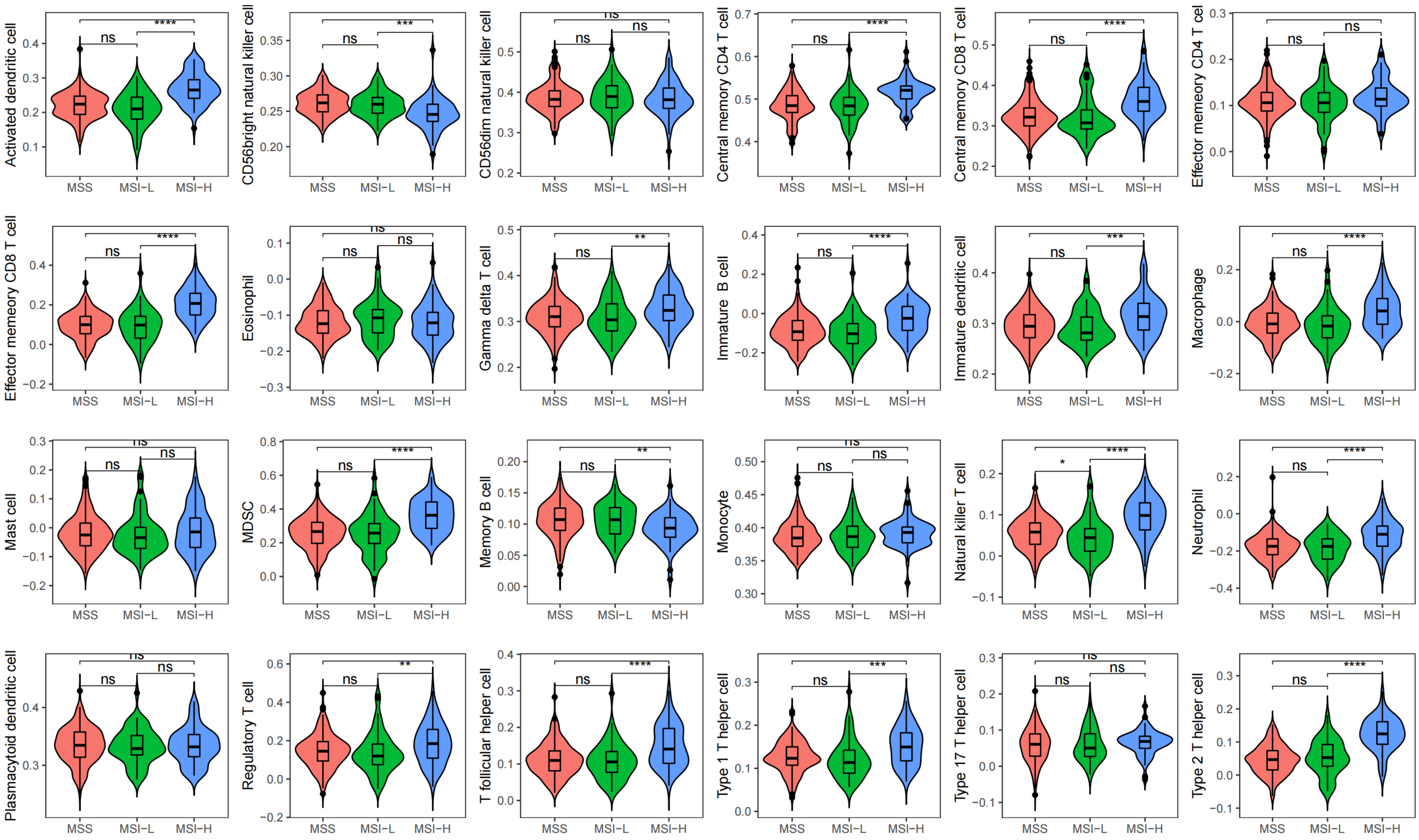

Figure S2. ssGESA analysis showing most of the 28 types of immune cell were evaluated in MSI-H sample except for CD56bright natural killer cell

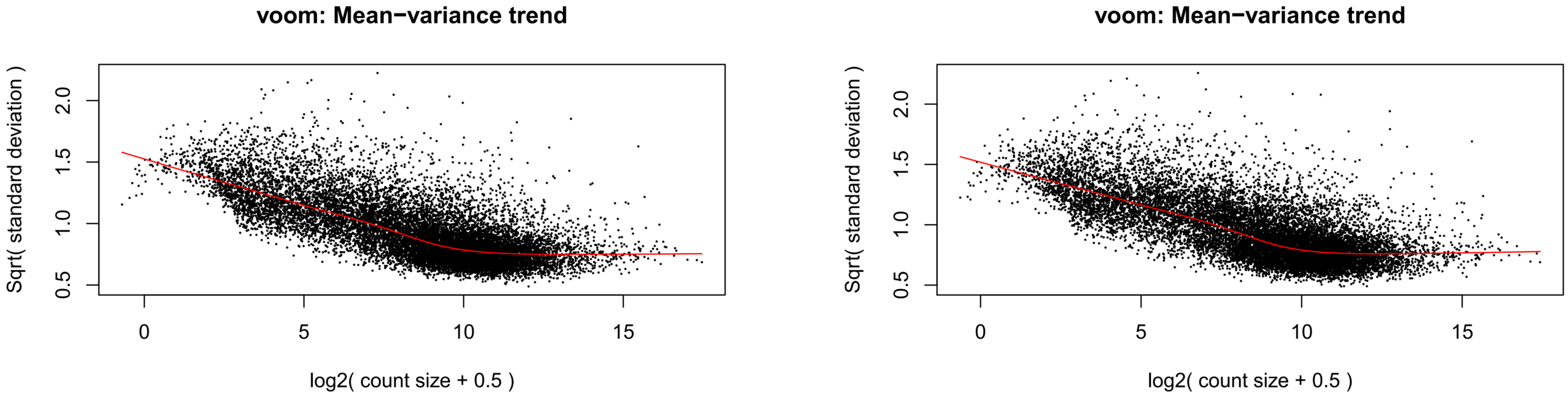

Figure S3. Voom normalization of (A) MSI-H vs MSS and MSI-H vs MSI-L

Gnen	Coefficients	Gnen	Coefficients	Gnen	Coefficients
CXCL13	-0.172536814	REG4	-0.010190369	ISM2	0.131214196
HOXC11	0.174511578	TNNT1	0.210607423	LRRN1	-0.22956863
HOXC9	0.42283998	CKMT2	-0.073719642	LY6G6E	0.575926646
NPSR1	0.20555483	CYP2W1	0.061827078	REN	0.082645245
ONECUT3	0.089662597	DEFA5	-0.014511923	SLC1A7	0.053515006
PLA2G2A	-0.064385888	DEFA6	-0.043513765	SLC3A1	0.152651502
REG1A	-0.00357952	EVX1	0.057065939	SOSTDC1	-0.001863137

Table S1. The 24 genes and corresponding coefficients determined by LASSO Cox regression
